# Sex disparities in the association between serum cotinine and chronic kidney disease

**DOI:** 10.18332/tid/185965

**Published:** 2024-04-29

**Authors:** Jianling Song, Ping Wang, Hong Li

**Affiliations:** 1 Department of Nephrology, The Second Affiliated Hospital, Jiangxi Medical College, Nanchang University, Nanchang, People's Republic of China; 2 Department of Gynecology and Obstetrics, Yongfeng People's Hospital, Jian, People's Republic of China; 3 Department of Medical Records, The Second Affiliated Hospital, Jiangxi Medical College, Nanchang University, Nanchang, People's Republic of China

**Keywords:** smoking, cotinine, chronic kidney disease, sex disparities

## Abstract

**INTRODUCTION:**

Despite the existence of numerous studies highlighting the adverse effects of smoking on kidney function, the investigation of the correlation between serum cotinine and chronic kidney disease (CKD) remains inconclusive due to insufficient evidence. Consequently, the primary objective of this study was to ascertain the association between serum cotinine levels and CKD.

**METHODS:**

This study analyzed data from 10900 Americans participating in the National Health and Nutrition Examination Survey between 2005 and 2016. The independent variable under investigation was log serum cotinine, while the dependent variable was the presence of CKD. To investigate the potential linear and non-linear correlations between serum cotinine and CKD, logistic regression models and generalized additive models (GAM) were employed. Furthermore, stratified analyses and interaction tests were conducted to evaluate potential disparities in the relationship between serum cotinine and CKD, based on sex.

**RESULTS:**

The median age in the study participants was 49.28 ± 17.96 years, and the median log serum cotinine (ng/mL) was -0.54 ± 1.68. The prevalence of CKD was found to be 17.04%. Multifactorial regression analysis did not show a statistically significant association between log serum cotinine and CKD (OR=1.02; 95% CI: 0.98–1.06, p=0.4387). A statistically significant non-linear association between log serum cotinine and CKD was also not observed in the GAM analysis (p non-linear value=0.091). Subgroup analyses revealed sex differences in the association between log serum cotinine and CKD. Briefly, males had a positive association between log serum cotinine and incident CKD (OR=1.08; 95% CI: 1.02–1.15, p=0.0049). In females, there was a U-shaped association between log serum cotinine and CKD, with an optimal inflection point for log serum cotinine of -0.30 (serum cotinine=0.5 ng/mL).

**CONCLUSIONS:**

Cross-sectional analyses of NHANES data showed gender differences in the association between serum cotinine and the development of CKD.

## INTRODUCTION

Chronic kidney disease (CKD) has a significant impact on a substantial portion of the adult population worldwide, affecting approximately 15–20% of individuals^1^. This condition is associated with an increased susceptibility to cardiovascular diseases and mortality^1,2^. As a significant global public disease, CKD has increased as a cause of death (from 25th in 1990 to 17th in 2015) and now accounts for 1.35% of the global burden of years of life lost with disability and is growing at a rate of 1% per year^3,4^. The management of CKD necessitates long-term dialysis and medication, imposing a substantial financial burden on patients^5,6^. The substantial incidence of CKD and its associated mortality rates, coupled with the exorbitant expenses incurred in medical care, impose a significant burden on individuals, families, and the healthcare system at large. Consequently, it becomes imperative to prioritize prevention and treatment strategies that are rooted in the underlying pathological mechanisms of CKD, as they hold the potential to mitigate CKD-related complications and fatalities.

Numerous studies have provided evidence of the detrimental effects of smoking on kidney function^7,8^. However, it is essential to acknowledge that the reliance on self-reported smoking data may introduce challenges in accurately recalling the quantity and duration of cigarette consumption, potentially resulting in imprecise assessments of smoking exposure^9,10^. To address this limitation, serum cotinine, a reliable indicator of secondhand smoke exposure^11^, was employed as a surrogate measure for smoking in the present investigation. Cotinine, the primary metabolite of nicotine in humans, predominantly resides in the bloodstream and possesses a prolonged half-life (3–4 days), rendering it a widely utilized marker for tobacco exposure^12^. Yi-Cheng et al.^13^. Investigated the relationship between serum cotinine and eGFR, revealing a negative correlation with eGFR. Similarly, Kataria et al.^14^ observed that elevated levels of serum cotinine (>2 ng/mL) were linked to increased creatinine and decreased eGFR. However, the relationship between serum cotinine and CKD remains unclear. This cross-sectional study, therefore, fills this gap by analyzing the effect of serum cotinine on CKD using National Health and Nutrition Examination Surveys (NHANES) data.

## METHODS

### Study population

The NHANES, a cross-sectional nationwide survey of the non-institutionalized population in the United States, is conducted by the National Center for Health Statistics (NCHS), a division of the American Centers for Disease Control and Prevention. This survey collects comprehensive data on demographics, socioeconomic indicators, personal habits, specific medical conditions, health concerns, and laboratory markers. The data collection process employs a stratified, multiple-stage probability cluster design. The NHANES statistics are released to the public biennially.

**Figure 1:**
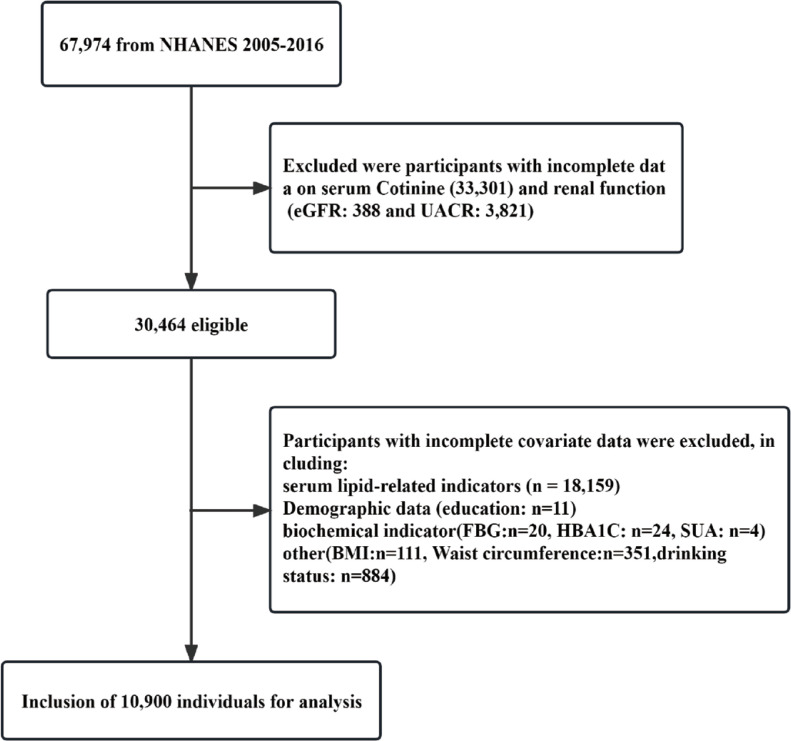
Flowchart for inclusion of participants, NHANES 2005–2016 (N=10900)

The NHANES survey conducted between 2005 and 2016 had a sizeable participation of 67974 individuals. Participants with incomplete data, specifically regarding renal function [urinary albumin to creatinine ratio (UACR): n=3821; estimated glomerular filtration rate (eGFR)=388], serum cotinine (n=33301), and other covariates (n=19564) were excluded from the study. Further information can be found in Figure 1. The project received approval from the Institutional Review Board of NCHS and was conducted by the Declaration of Helsinki^15^. Each participant provided informed consent by completing a consent form.

### Outcome measurements

The primary outcome indicator in this study was CKD, which was diagnosed based on an eGFR of <60 mL/min/1.73m^2^ or a UACR of 30 mg/g or higher^16^. The UACR was measured using randomly collected urine samples, while albumin levels were determined by analyzing spot urine samples using a solid-phase fluorescence immunoassay^17^. Urinary creatinine levels were measured using the enzyme technique (creatinase) on a Roche/Hitachi Modular P chemistry analyzer. The eGFR was calculated using the CKD-Epidemiology Collaborative (CKD-EPI) formula^18^.

### Exposure measurements

Serum cotinine concentrations were obtained from the NHANES database using isotope dilution highperformance liquid chromatography/atmospheric pressure chemical ionization tandem mass spectrometry^19^. Given the non-normal distribution of serum cotinine, a log 10 transformation was conducted.

### Covariates

Demographic data, encompassing sex, age, race, poverty income ratio (PIR), and education level, were acquired from the NHANES questionnaire. Participants were requested to respond to the abovementioned inquiries at their discretion. Furthermore, hematological indicators, comprising fasting blood glucose (FBG), glycosylated hemoglobin A1c (HBA1C), serum uric acid (SUA), triglycerides (TG), total cholesterol (TC), high-density lipoprotein cholesterol (HDL), and low-density lipoprotein cholesterol (LDL), were exclusively obtained from the NHANES database by extracting participants’ blood samples and dispatching them to a specialized laboratory for analysis. Waist circumference, weight, and height were determined through physical examination. Body mass index (BMI) was calculated as the ratio of an individual’s weight in kilograms to their height in meters squared.

Data on alcohol and tobacco usage was collected through the administration of questionnaires. Participants were queried about their alcohol consumption within the preceding 12 months, categorizing them as non-drinkers if they responded with ‘Never’ and as drinkers otherwise. Likewise, participants were asked about their current cigarette smoking habits to determine their smoking history. Respondents who answered affirmatively were classified as smokers, whereas those who responded negatively were classified as non-smokers.

The medical history of the participants was obtained through the utilization of a survey and physical examination. The identification of hypertension was established by inquiring about the participants’ history of hypertension, their use of medication for hypertension, or by measuring their blood pressure on three occasions (with systolic readings ≥140 mm Hg or diastolic readings ≥90 mm Hg). Likewise, the diagnosis of diabetes was ascertained by inquiring about the participants’ history of diabetes or their use of medication to regulate blood glucose levels. It is crucial to acknowledge that participants’ data about the utilization of prescription medications was solely acquired through self-reports.

### Statistical analysis

The baseline characteristics present continuous variables as means and standard deviations for normal distribution or medians for the interquartile range. Categorical variables are presented as percentages. Kruskal-Wallis rank sum test and Fisher’s exact probability test were used to analyze differences between multiple groups. The adjustment variables for the multifactorial logistic regression analysis model were chosen based on their impact on the log serum cotinine regression coefficient, with a threshold of >10%, or if the covariate had a regression coefficient of CKD with a p<0.1.

Logistic regression analyses log the odds ratio (OR) and 95% confidence interval (CI) of the association between serum cotinine and CKD. Three models were fitted in this study; model 1 was unadjusted; model 2 was adjusted for age, sex, race, poverty income ratio, and education level; model 3 was further adjusted for FBG, HBA1C, SUA, TG, LDL, BMI, waist circumference, diabetes, hypertension, smoking, drinking, glucose-lowering drugs, hypolipidemic drugs, antihypertensive drugs.

A generalized additive model (GAM) was used to test the non-linear relationship between CKD incidence and log serum cotinine, and the nonlinear results were presented using a smoothed curve fit plot. A two-piece linear regression model with a smoothing function was used to investigate the threshold effect of log serum cotinine on CKD incidence. Thresholds, or inflection points, were identified by iterative testing in which inflection points were selected at predetermined intervals, followed by a selection of inflection points that maximized the likelihood of the model. In addition, a log-likelihood ratio test was performed to compare the fit of a single linear regression model with that of a two-segment linear model. Sex-specific differences in the association between serum cotinine and CKD were evaluated through stratified analyses and interaction tests. The statistical software R Foundation (http://www.R-project.org) and EmpowerStats (http://www.empowerstats.com, X&Y Solutions, Inc., Boston, MA) were utilized for all analyses. The predetermined level of statistical significance was set at p<0.05.

## RESULTS

### Baseline characteristics of participants

This study encompassed a sample size of 10900 individuals, consisting of 5492 females and 5408 males. The median age was determined to be 49.28 ± 17.96 years, while the median log serum cotinine was found to be -0.54 ± 1.68. The prevalence of CKD was observed to be 17.04%. The demographic and clinical attributes of the three subgroups, categorized based on log serum cotinine, are presented in Table 1. Participants in the high-log serum cotinine group exhibited characteristics such as being younger, male, non-Hispanic white, smokers, and alcohol drinkers, in comparison to participants in the low-log serum cotinine group. Furthermore, the high-log serum cotinine group displayed elevated levels of SUA, TG, TC, and eGFR while exhibiting lower levels of PIR, UACR, HDL, and BMI. Additionally, the high-log serum cotinine group demonstrated a decreased prevalence of hypertension, diabetes, and CKD, along with lower rates of taking hypoglycemic, antihypertensive, and lipid-lowering medications. However, the statistical significance of FBG, HBA1C, and LDL was not observed in the group with high log serum cotinine levels.

**Table 1:** Baseline characteristics and demographics of the study population based on log serum cotinine tertiary subgroups, NHANES 2005–2016 (N=10900)

*Characteristics*	*Log serum cotinine (ng/mL)*			
	*Low (-1.96 to < -1.72) n (%)*	*Medium (-1.72 to < -0.60) n (%)*	*High (-0.60 to 3.2) n (%)*	*p*
**Total**, n	3582	3682	3636	
**Age** (years), mean ± SD	52.91 ± 17.99	49.96 ± 18.11	45.01 ± 16.85	<0.001
**Sex**				<0.001
Female	2088 (58.29)	1906 (51.77)	1498 (41.20)	
Male	1494 (41.71)	1776 (48.23)	2138 (58.80)	
**Race**				<0.001
Non-Hispanic White	1579 (44.08)	1453 (39.46)	1722 (47.36)	
Non-Hispanic Black	387 (10.80)	738 (20.04)	983 (27.04)	
Mexican American	754 (21.05)	582 (15.81)	362 (9.96)	
Other	862 (24.06)	909 (24.69)	569 (15.65)	
**Education level**				<0.001
Junior high and high school	1347 (37.60)	1679 (45.60)	2241 (61.63)	
College	2235 (62.40)	2003 (54.40)	1395 (38.37)	
**Measurements**, mean ± SD				
PIR (%)	2.94 ± 1.63	2.57 ± 1.61	2.00 ± 1.50	<0.001
FBG (mmol/L)	6.02 ± 1.82	6.07 ± 1.93	5.97 ± 1.88	0.107
HBA1C (%)	5.76 ± 1.01	5.76 ± 1.06	5.74 ± 1.10	0.542
SUA (μmol/L)	318.17 ± 80.57	331.77 ± 85.07	333.12 ± 85.26	<0.001
TG (mmol/L)	1.31 ± 0.72	1.30 ± 0.74	1.38 ± 0.77	<0.001
TC (mmol/L)	4.99 ± 1.05	4.93 ± 1.03	4.92 ± 1.06	0.025
HDL (mmol/L)	1.45 ± 0.41	1.40 ± 0.39	1.35 ± 0.42	<0.001
LDL (mmol/L)	2.94 ± 0.90	2.94 ± 0.89	2.94 ± 0.94	0.945
eGFR (mL/min/1.73m^2^)	91.87 ± 23.26	94.47 ± 24.04	99.11 ± 22.41	<0.001
BMI (kg/m^2^)	28.76 ± 6.36	29.33 ± 6.82	28.65 ± 6.98	<0.001
Waist circumference (cm)	98.48 ± 15.60	99.49 ± 16.31	98.81 ± 16.97	0.027
UACR (mg/g), median (IQR)	7.42 (4.76–14.00)	6.94 (4.54–13.08)	6.82 (4.31–13.88)	0.154
**Diabetes**				<0.001
Yes	537 (14.99)	500 (13.58)	412 (11.33)	
No	3045 (85.01)	3182 (86.42)	3224 (88.67)	
**Hypertension**				<0.001
Yes	1510 (42.16)	1597 (43.37)	1421 (39.08)	
No	2072 (57.84)	2085 (56.63)	2215 (60.92)	
**CKD**				0.005
Yes	642 (17.92)	656 (17.82)	559 (15.37)	
No	2940 (82.08)	3026 (82.18)	3077 (84.63)	
**Smoking**				<0.001
Yes	968 (27.02)	1107 (30.07)	2768 (76.13)	
No	2614 (72.98)	2575 (69.93)	868 (23.87)	
**Drinking**				<0.001
Yes	2869 (80.09)	3006 (81.64)	3390 (93.23)	
No	713 (19.91)	676 (18.36)	246 (6.77)	
Glucose-lowering drugs	465 (12.99)	419 (11.39)	349 (9.60)	<0.001
Hypolipidemic drugs	895 (25.00)	745 (20.25)	589 (16.20)	<0.001
Antihypertensive drugs	248 (6.93)	277 (7.53)	188 (5.17)	<0.001

FBG: fasting blood glucose. UACR: urinary albumin to creatinine ratio. eGFR: estimated glomerular filtration rate. SUA: serum uric acid. BMI: body mass index. TC: total cholesterol. TG: triglycerides. LDL: low-density lipoprotein cholesterol. HDL: high-density lipoprotein cholesterol. HbA1c: glycosylated hemoglobin A1c. FBG: fasting blood glucose. IPR: poverty income ratio. CKD: chronic kidney disease. IQR: interquartile range.

### No significant association was found between log serum cotinine and CKD prevalence

Logistic regression analyses showed that after adjustment for age, sex, race, PIR, education level, FBG, HBA1C, SUA, TG, LDL, BMI, waist circumference, diabetes, hypertension, smoking, drinking, glucose-lowering drugs, antihypertensive drugs, glucose-lowering drugs, hypolipidemic drugs, and antihypertensive drugs, log serum cotinine was associated with a 2% correlation with incident CKD in the overall population. However, this association did not reach statistical significance (p>0.05) (Table 2). To evaluate the association between log serum cotinine and CKD, log serum cotinine was divided into three equal segments, with the low-log serum cotinine group serving as the reference group. The relationship between log serum cotinine and CKD was examined while controlling for various demographic and health-related factors, including age, sex, race, PIR, education level, FBG, HBA1C, SUA, TG, LDL, BMI, waist circumference, diabetes, hypertension, smoking, drinking, glucose-lowering drugs, hypolipidemic drugs, antihypertensive drugs. The results showed that the likelihood of developing CKD increased by 4% and 6% in the medium-log serum cotinine group and the high-log serum cotinine group, respectively, compared to the low-log serum cotinine group (Table 2). However, this difference was not statistically significant (p>0.05). The above results indicate that there is no statistically significant linear relationship between log serum cotinine and CKD.

**Table 2:** Relative odds of chronic kidney disease according to log serum cotinine in different models among all participants, NHANES 2005–2016 (N=10900)

*Log serum cotinine (ng/mL)*	*Model 1*		*Model 2*		*Model 3*	
	*OR (95% CI)*	*p*	*AOR (95% CI)*	*p*	*AOR (95% CI)*	*p*
Per 1 increment	0.95 (0.92–0.98)	0.0004	1.02 (0.98–1.06)	0.3908	1.02 (0.98–1.06)	0.4387
Low (-1.96 to < -1.72) ®	1		1		1	
Medium (-1.72 to < -0.60)	0.99 (0.88–1.12)	0.9057	1.11 (0.96–1.27)	0.1526	1.04 (0.90–1.20)	0.5953
High (-0.60 to 3.2)	0.83 (0.73–0.94)	0.0037	1.11 (0.95–1.29)	0.1859	1.06 (0.90–1.25)	0.4777
p for trend	0.0011		0.3740		0.3037	

AOR: adjusted odds ratio. Model 1: unadjusted. Model 2 was adjusted for age, sex, race, poverty income ratio, and education level. Model 3 was adjusted as for Model 2 but also for fasting blood glucose, glycosylated hemoglobin A1c, serum uric acid, triglycerides, low-density lipoprotein cholesterol, body mass index, waist circumference, diabetes, hypertension, smoking, drinking, glucose-lowering drugs, hypolipidemic drugs, and antihypertensive drugs. ® Reference category.

**Figure 2:**
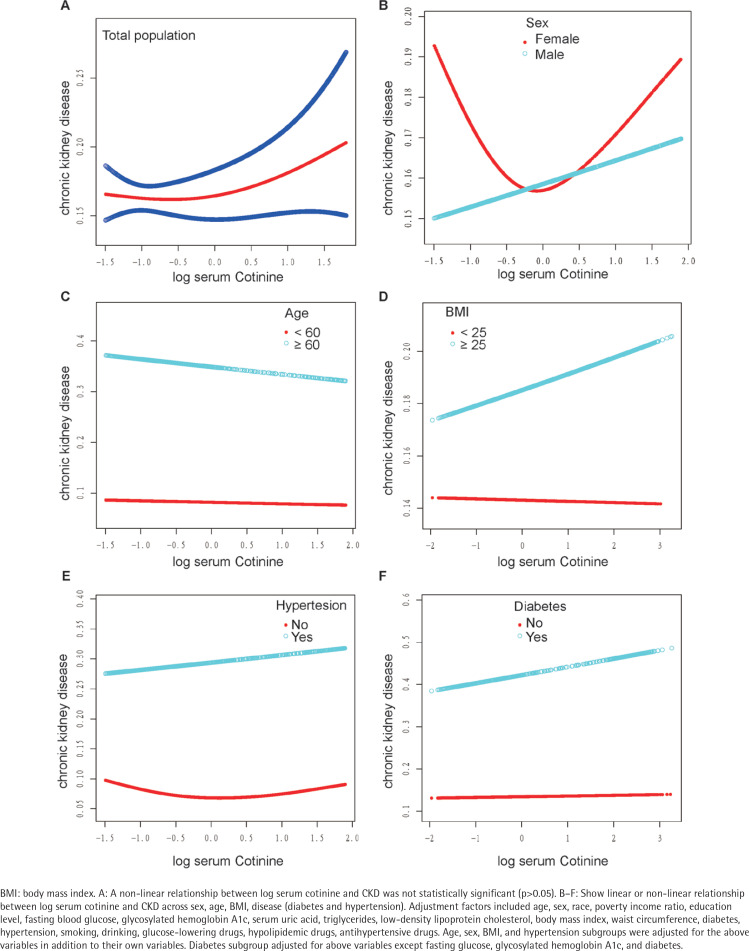
Association between log serum cotinine and the prevalence of chronic kidney disease (CKD) in total population and different subgroups (red and blue lines represent the estimated values and their corresponding 95% confidence intervals), NHANES 2005–2016 (N=10900)

Furthermore, an examination was conducted to evaluate the non-linear association between log serum cotinine and CKD. The analysis revealed a U-shaped correlation between log serum cotinine and CKD in the overall population, as depicted in Figure 2A. Subsequently, the inflection point was determined using the threshold effect, as presented in Table 3. In summary, when log serum cotinine is below 1.28 ng/mL, a one ng/mL increment in log serum cotinine leads to a 2% reduction in the likelihood of developing CKD. Conversely, when log serum cotinine exceeds 1.28 ng/mL, a one ng/mL increase in log serum cotinine amplifies the odds of developing CKD by a factor of 3.93.

**Table 3:** Threshold effect of log serum cotinine on the chronic kidney disease analyzed using piece-wise linear regression in total population and female/male population, NHANES 2005–2016 (N=10900)

*Log serum cotinine (ng/mL)*	*Chronic kidney disease*		
	*AOR (95% CI)*	*p*	*p non-linear value (p for log-likelihood ratio test)*
**Total population^a^**			0.091
<1.28	0.98 (0.83–1.14)	0.7550	
≥1.28	3.93 (0.87–17.66)	0.0744	
**Females^b^**			0.034
< -0.30	0.63 (0.42–0.95)	0.0275	
≥ -0.30	1.28 (0.91–1.80)	0.1496	
**Males^b^**			0.062
<1.63	1.16 (0.97–1.40)	0.1095	
≥1.63	0.00 (0.00–2.30)	0.0900	

a Adjusted for age; sex, race, poverty income ratio, education level, fasting blood glucose, glycosylated hemoglobin A1c, serum uric acid, triglycerides, low-density lipoprotein cholesterol, body mass index, waist circumference, diabetes, hypertension, smoking, drinking, glucose-lowering drugs, hypolipidemic drugs, and antihypertensive drugs.

b Adjusted for all of the above variables except sex.

### The relationship between log serum cotinine and CKD varies based on sex

Figures 2B–2F displays the correlation between log serum cotinine and CKD in a diverse population, considering sex, age, BMI, diabetes, and hypertension. Notably, sex differences were observed. In the fully adjusted model, a U-shaped relationship between log serum cotinine and CKD was observed in females. Conversely, a linear relationship was observed in males. This finding was further supported by the results presented in Table 4, which indicated that as the log serum cotinine value increased, males were more likely to be at risk for CKD (p for interaction=0.0295). Specifically, a one ng/mL increase in log serum cotinine was associated with an 8% increase in the risk of developing CKD in males. The incidence of CKD exhibited a notable elevation in both the medium-log serum cotinine group and the high-serum cotinine group compared to the low-log serum cotinine group (p for trend=0.0014). Furthermore, our analysis revealed a critical threshold for log serum cotinine at -0.3 ng/mL in females, specifically serum cotinine at 0.50 ng/mL (Table 3). In instances where log serum cotinine is below -0.30, an augmentation of 1 ng/mL in log serum cotinine is linked to a 37% reduction in the likelihood of developing CKD. Conversely, when log serum cotinine surpasses -0.30, a one ng/mL increase in log serum cotinine is associated with a 1.28-fold increase in the odds of developing CKD. Furthermore, no non-linear associations were detected among age (<60, ≥60), BMI (<25, ≥25) , diabetes (yes/no), and hypertension (yes/no). Finally, we also examined the linear relationship between log serum cotinine (continuous/trivial) and CKD in different populations. There was no statistically significant linear relationship between log serum cotinine and CKD in different genders (<60, ≥60), BMI (<25, ≥25) , hypertensive/non-hypertensive and diabetic/non-diabetic populations (p>0.05) (Supplementary file Table 1 and Figure 1).

**Table 4:** Relative odds of chronic kidney disease according to log serum cotinine in different models among males and females, NHANES 2005–2016 (N=10900)

*Log serum cotinine (ng/ mL)*	*Model 1*		*Model 2*		*Model 3*	
	*OR (95% CI)*	*p*	*AOR (95% CI)*	*p*	*AOR (95% CI)*	*p*
**Females**						
Per 1 increment	0.95 (0.90–0.99)	0.0197	0.98 (0.92–1.03)	0.3678	0.96 (0.91–1.02)	0.2189
Low (-1.96 to < -1.74) ®	1		1		1	
Medium (-1.92 to < -0.60)	1.06 (0.91–1.24)	0.4646	1.10 (0.92–1.32)	0.3052	0.99 (0.82–1.20)	0.9335
High (-0.60 to 3.2)	0.85 (0.71–1.02)	0.0736	0.93 (0.75–1.15)	0.4950	0.85 (0.67–1.06)	0.1462
p for trend	0.0267		0.2556		0.2223	
**Males**						
Per 1 increment	0.95 (0.91–0.99)	0.0264	1.06 (1.01–1.12)	0.0283	1.08 (1.02–1.15)	0.0049
Low (-1.96 to < -1.74) ®	1		1		1	
Medium (-1.92 to < -0.60)	0.93 (0.77–1.11)	0.4095	1.13 (0.91–1.39)	0.2694	1.14 (0.91–1.43)	0.2518
High (-0.60 to 3.2)	0.83 (0.70–0.99)	0.0431	1.33 (1.07–1.67)	0.0116	1.42 (1.11–1.80)	0.0044
p for trend	0.0506		0.0151		0.0014	
p value for interaction	0.8263		0.0300		0.0295	

AOR: adjusted odds ratio. Model 1: unadjusted. Model 2 was adjusted for age, sex, race, poverty income ratio, and education level. Model 3 was adjusted as for Model 2 but also for fasting blood glucose, glycosylated hemoglobin A1c, serum uric acid, triglycerides, low-density lipoprotein cholesterol, body mass index, waist circumference, diabetes, hypertension, smoking, drinking, glucose-lowering drugs, hypolipidemic drugs, and antihypertensive drugs. ® Reference category.

## DISCUSSION

This research is a thorough analysis of NHANES data from 2005 to 2016. Our results show that having higher log serum cotinine levels is correlated with an increased risk of developing CKD, but it’s important to note that this correlation did not reach statistical significance. Further investigation into the non-linear relationship between log serum cotinine and CKD revealed a U-shaped pattern that lacked statistical significance. Subgroup analyses examined the association between log serum cotinine and CKD in various populations and identified a specific U-shaped correlation among females. An inflection point of 0.50 ng/mL serum cotinine was identified in this subgroup analysis. Furthermore, our study revealed a significant correlation between the log serum cotinine levels and the incidence of CKD, specifically in males.

Smoking is an independent risk factor for incident CKD, as has been confirmed in several studies^20-22^. Smoking has been found to induce a decline in kidney function^23-25^. However, research examining the relationship between cotinine and CKD has been scarce. Two different research studies have explored the link between urinary cotinine levels and kidney function. In one study conducted by Charlotte et al. ^26^, the relationship between urinary cotinine and renal function was examined in a population with CKD. The findings revealed a weak correlation between urinary cotinine and eGFR (R=0.40, p=0.005). However, no significant association was observed between urinary cotinine and 24-hour urinary protein excretion. Another study conducted as part of a Korean national survey^27^ reported that individuals confirmed as smokers based on urinary cotinine levels were significantly more likely to exhibit moderately elevated urinary albumin (OR=4.37; 95% CI: 1.63–11.71). Furthermore, the U.S. NHANES study investigated the association between serum cotinine and eGFR, revealing a significant negative correlation between the two variables^13^. Additionally, the study observed a non-linear relationship between serum cotinine and eGFR when analyzed by sex^13^.

This cross-sectional study identified a lack of statistical significance in the relationship between log serum cotinine levels and the heightened likelihood of developing CKD after accounting for confounding factors. Furthermore, a non-statistically significant U-shaped association between log serum cotinine levels and the risk of CKD development was observed. Additionally, a statistically significant U-shaped association between log serum cotinine levels and outcome events was found exclusively in females but not males. We postulate that this discrepancy may be attributed to variations in nicotine and cotinine metabolism influenced by sex hormones^28^. Further exploration of more in-depth mechanisms is still required.

When considering the existing literature, it is evident that smoking has a detrimental impact on renal function. Smoking can be attributed to the various effects of smoking and nicotine on sympathetic activation, endothelial impairment, and systemic hemodynamics. These factors ultimately result in significant changes in renal hemodynamics and albuminuria^29^. Ritz et al.^30^ provide additional evidence to support the above claim, revealing a positive correlation between smoking and heightened mean arterial pressure, heart rate, and elevated arginine vasopressin and adrenaline levels. Moreover, individuals who engage in smoking consistently experience a decline in glomerular filtration rate, accompanied by a significant decrease in filtration fraction and an increase in neovascular resistance^30^. There are various non-hemodynamic factors, such as oxidative stress, reduced nitric oxide bioavailability, elevated endothelin one concentration, tubular cell injury, and decreased angiotensin-converting enzyme that contribute to the detrimental impact of smoking on renal function^31^. Furthermore, the exposure to smoke resulted in a notable augmentation of fibrosis markers in the renal tissues of a mouse model with CKD. Additionally, the CKD model mice exhibited a substantial elevation in systolic blood pressure, renal fibrosis, and a decline in renal function, potentially attributed to the reduced expression of miR-29b-3p^32^. Moreover, nicotine directly induces a reduction in synaptic podoplanin in glomerular podocytes in a mouse model of diabetes, leading to an increase in proteinuria and fibronectin expression in a subset of individuals with diabetes^33^. Significantly, the act of smoking is associated with an elevated likelihood of all-cause mortality and cardiovascular fatality among individuals diagnosed with CKD^34,35^. Moreover, the cessation of smoking has been found to diminish proteinuria notably, impede the advancement towards end-stage renal disease, and mitigate the risk of mortality in patients with CKD and diabetes, thus emphasizing the importance of motivating smokers to abandon this habit promptly^36,37^. Formanek et al.^35^ provided valuable insights on effective strategies to aid individuals in their smoking cessation endeavors. The fundamental reality is that smokers can attain smoking cessation by employing a combination of behavioral interventions and pharmacologic therapies. Our research has yielded a more precise numerical value specifically for females, thereby informing them that an elevation in serum cotinine levels augments the likelihood of CKD, even in the absence of active smoking.

### Limitations

This study has some limitations. Primarily, this study exhibits a restricted capacity to investigate and validate etiologic hypotheses and extrapolations. Further cohort studies are necessary to corroborate the association between serum cotinine and CKD. Furthermore, this study necessitated the exclusion of a substantial number of participants due to incomplete data, potentially compromising its representativeness of the US population. Moreover, considering the disparities between nations, the generalizability of the study’s findings to other countries remains to be determined. In addition, data on prescription drug use and alcohol and tobacco use in this study were obtained from participants’ self-reports. The accuracy of self-reported data depends on the accuracy of each self-reported variable, and we did not specifically examine the reliability of self-reported data for these variables. Lastly, the presence of confounding variables that are unidentified or unpredictable cannot be entirely ruled out.

## CONCLUSIONS

This cross-sectional study was carried out on a sample of 10900 American individuals, revealing a lack of statistical significance in the linear and non-linear association between log serum cotinine levels and the occurrence of CKD. Additionally, our findings demonstrated sex-specific variations in the relationship between serum cotinine and CKD. Specifically, a U-shaped relationship was observed in females, whereas no such pattern was observed in males. Sex differences are intrinsically inherent. Our investigation has identified disparities between serum cotinine and CKD based on sex, although the precise mechanism remains unknown. We postulate a potential influence of sex hormones on cotinine metabolism, necessitating further investigation.

## Supplementary Material



## Data Availability

The data supporting this research are available from the following source: https://www.cdc.gov/nchs/nhanes/index.htm
