# Supplementary material for: Sex disparities in the association between serum cotinine and chronic kidney disease
Source: Tob Induc Dis. 2024 Apr 29;22:10.18332/tid/185965. doi: 10.18332/tid/185965 (PMC11056949; doi:10.18332/tid/185965)
Supplement: Supplementary file 1 [file TID-22-68-s1.pdf]

Supplementary Table 1 Logistic regression analysis of the association between log serum cotinine (continuous/trivial) and Chronic kidney disease in different subgroups (NHANES 2005-2016, N=10,900)

**Log serum cotinine (ng/mL)**	**Chronic kidney disease OR (95%CI) P value**		
**Age**	<60	≥60	Total
**Per 1 increment**	1.05 (0.98, 1.13) 0.1666	0.99 (0.93, 1.05) 0.7374	1.00 (0.95, 1.04) 0.9218
**Log serum cotinine tertile**			
**Low (-1.96 to < -1.72)**	1.0	1.0	1.0
**Middle (-1.72 to < -0.60)**	0.99 (0.78, 1.26) 0.9457	1.02 (0.85, 1.23) 0.8121	1.01 (0.87, 1.17) 0.9014
**High (-0.60 to 3.2)**	1.09 (0.83, 1.44) 0.5311	0.96 (0.77, 1.21) 0.7396	0.97 (0.82, 1.16) 0.7575
**BMI**	<25	≥25	
**Per 1 increment**	1.01 (0.92, 1.10) 0.8416	1.04 (0.99, 1.10) 0.1204	1.03 (0.98, 1.07) 0.2263
**Log serum cotinine tertile**			
**Low (-1.96 to < -1.72)**	1.0	1.0	1.0
**Middle (-1.72 to < -0.60)**	1.09 (0.81, 1.47) 0.5583	1.04 (0.88, 1.23) 0.6236	1.05 (0.90, 1.21) 0.5495
**High (-0.60 to 3.2)**	1.12 (0.78, 1.62) 0.5410	1.11 (0.91, 1.35) 0.3005	1.09 (0.92, 1.30) 0.3068
**Hypertension**	No	Yes	Total
**Per 1 increment**	1.00 (0.93, 1.08) 0.9574	1.07 (1.01, 1.14) 0.0155	1.03 (0.99, 1.08) 0.1791
**Log serum cotinine tertile**			
**Low (-1.96 to < -1.72)**	1.0	1.0	1.0
**Middle (-1.72 to < -0.60)**	1.22 (0.96, 1.56) 0.1076	1.00 (0.83, 1.20) 0.9781	1.04 (0.90, 1.21) 0.5593
**High (-0.60 to 3.2)**	0.99 (0.74, 1.32) 0.9290	1.28 (1.02, 1.60) 0.0298	1.11 (0.93, 1.31) 0.2574
**Diabetes**	No	Yes	
**Per 1 increment**	1.02 (0.97, 1.07) 0.4417	1.07 (0.98, 1.17) 0.1451	1.03 (0.99, 1.08) 0.1791
**Log serum cotinine tertile**			
**Low (-1.96 to < -1.72)**	1.0	1.0	1.0
**Middle (-1.72 to < -0.60)**	1.06 (0.89, 1.25) 0.5165	1.04 (0.78, 1.41) 0.7741	1.04 (0.90, 1.21) 0.5593
**High (-0.60 to 3.2)**	1.08 (0.88, 1.32) 0.4820	1.21 (0.86, 1.70) 0.2753	1.11 (0.93, 1.31) 0.2574

Adjustment factors included age; sex; race; poverty income ratio; education; fasting blood glucose; glycosylated hemoglobin A1c; serum uric acid; triglycerides; low-density lipoprotein cholesterol; body

mass index; waist circumference; diabetes; hypertension; smoking; drinking; glucose-lowering drugs; hypolipidemic drugs; antihypertensive drugs.
Age, BMI, and hypertension subgroups were adjusted for the above variables in addition to their own variables.
Diabetes subgroup adjusted for above variables except fasting glucose, glycosylated hemoglobin A1c and diabetic.

Supplementary Figure 1

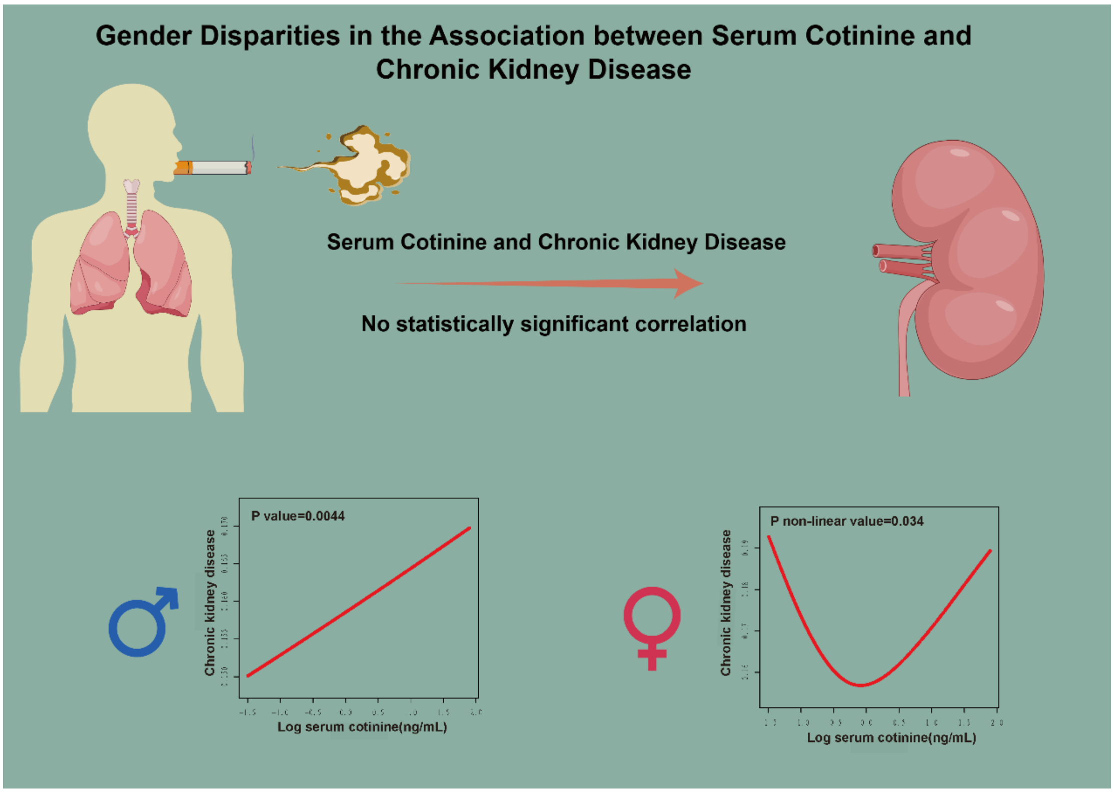

© 2024 Song J. et al.
